# Incidence and predictors of treatment-related mortality in paediatric acute leukaemia in El Salvador

**DOI:** 10.1038/sj.bjc.6604895

**Published:** 2009-03-17

**Authors:** S Gupta, M Bonilla, S L Fuentes, M Caniza, S C Howard, R Barr, M L Greenberg, R Ribeiro, L Sung

**Affiliations:** 1 Division of Haematology/Oncology and Program in Child Health Evaluative Sciences, The Hospital for Sick Children, 555 University Avenue, Toronto, ON, Canada M5G1X8; 2 Pediatric Oncology, Benjamin Bloom National Children's Hospital, San Salvador, El Salvador; 3 Pediatric Hematology/Oncology, St. Jude Children's Research Hospital, 262 Danny Thomas Place, Memphis, TN 38105-3678, USA; 4 Department of Infectious Diseases, St Jude Children's Research Hospital, Memphis, TN 38105-3678, USA; 5 Pediatric Hematology/Oncology, McMaster University, 1200 Main Street West, Hamilton, ON, Canada L8N 3Z5

**Keywords:** acute lymphoblastic leukaemia, acute myeloid leukaemia, treatment-related death, developing countries

## Abstract

Survival rates among children with leukaemia in low-income countries are lower than those in high-income countries. This has been attributed in part to higher treatment-related mortality (TRM). We examined the demographics, treatment, and outcomes of paediatric patients in El Salvador with acute lymphoblastic leukaemia (ALL) or acute myeloid leukaemia (AML) to determine the incidence, causes, and risk factors for TRM. Two trained data managers collected data prospectively; no patients were excluded. Biological, socioeconomic and nutritional predictors were examined. A total of 469 patients with ALL and 78 patients with AML were included. The 2-year cumulative incidence of TRM was significantly higher among children with AML (35.4±6.4%) than those with ALL (12.5±1.7%; *P*<0.0001). However, the proportion of deaths attributable to the toxicity of treatment did not differ significantly between AML (25/47, 53.2%) and ALL (55/107, 51.4%; *P*=0.98). Among children with ALL, low monthly income (*P*=0.04) and low parental education (*P*=0.02) significantly increased the risk of TRM. Among children with AML, biological, socioeconomic, and nutritional variables were not associated with TRM. In this low-income country, toxic death significantly contributes to mortality in both ALL and AML. A better understanding of the effect of socioeconomic status on TRM may suggest specific strategies for patients with ALL.

The past four decades have seen steady improvements in treatment outcomes for children with leukaemia. In high-income countries (HICs), overall cure rates of approximately 80% have been achieved in acute lymphoblastic leukaemia (ALL) (Pui and Evans, 2006). Recent trials in children with acute myeloid leukaemia (AML) also have shown improved outcomes and have resulted in event-free survival (EFS) of approximately 50% (Stevens *et al*, 1998; Ravindranath *et al*, 2005; Creutzig *et al*, 2006). These advances have been attributed to more efficacious use of antileukaemic agents, intensification of treatment when appropriate, identification of prognostic factors for risk stratification, and improvements in supportive care (Ravindranath *et al*, 2005; Pui and Evans, 2006).

Unfortunately, these advances in survival have not fully translated into low-income countries (LICs) where EFS is significantly lower than in HICs. Potential reasons for different survival rates include a higher rate of relapse, more abandonment of treatment, and higher rates of treatment-related mortality (TRM) in LICs. Improvements in outcome may be achieved by improving disease control, reducing abandonment, and reducing TRM. The latter becomes particularly important in LICs where TRM is an important contributor to poor outcomes.

The primary objectives of this study were to determine the incidence and causes of TRM among children with ALL and AML in El Salvador. The secondary objectives were to determine predictors of TRM in the same population and to describe TRM in El Salvador relative to published results in HICs and other LICs.

## Materials and methods

### Data source

Data were obtained from the Pediatric Oncology Networked Database (POND) (http://www.pond4kids.org). POND is an online database for paediatric cancer patient information designed to permit users at multiple locations to store and analyse data that include patient demographics, diagnoses, treatments, and outcomes in a secure environment with stringent control of access and privacy. Although the primary purpose of data collection is to monitor patient outcomes and implement quality improvement initiatives, data also can be extracted without personal identifiers for research. At the paediatric cancer centre in El Salvador, two data managers recorded information about patients and their outcomes in real time through chart abstraction. In El Salvador, as in 30 other countries, information about paediatric oncology patients is collected routinely, and includes data on patient demographics, nutrition, socioeconomic status, diagnosis, treatment, complications, and outcomes. A recent audit of POND data quality conducted in Honduras, where a similar data management program is in place, showed that accuracy for basic data fields was 99% (Ayoub *et al*, 2007).

### Study population

The patient sample consisted of children with ALL and AML treated at the Benjamin Bloom National Children's Hospital (San Salvador, El Salvador). This is the sole treatment centre for children with cancer in the country, in which approximately 200 new patients are treated a year. We included those aged 0–16 years at diagnosis who had *de novo* ALL or AML and were diagnosed between 1 January 2000 and 1 July 2007. Patients with acute promyelocytic and mature B-cell leukaemia were excluded.

Patients with ALL were treated according to the El Salvador-Guatemala-Honduras II protocol, which was based on the St Jude Total XIII (Pui *et al*, 2004b) and Total XV (Pui *et al*, 2004a) protocols. Significant modifications included the use of only two risk groups (lower and higher, Table 1), the omission of etoposide, and the administration of high-dose methotrexate as a 3-h infusion at a dose of 2 g m^−2^ for lower risk and 3 g m^−2^ for higher risk patients.

Between January 2000 and December 2006, patients with AML were treated according to the AHOPCA-AML 1999 protocol, which was based on BFM-AML93 (Creutzig *et al*, 2001). Significant modifications included the treatment of all children as low risk (without stem cell transplantation, which is unavailable in the region), reduction of daunorubicin by 66%, omission of etoposide during induction and intensification, omission of cyclophosphamide during consolidation, use of high-dose cytarabine and mitoxantrone only in patients without response after induction plus consolidation, and reduction of maintenance therapy to 12 months. After January 2007, patients with AML were treated with the AHOPCA-AML 2007 protocol, based on NOPHO-AML 93 (Lie *et al*, 2003). Significant modifications included induction therapy as per AHOPCA-AML 1999 (BFM-AML93), but with the addition of etoposide.

The oncology unit at the Benjamin Bloom Hospital has 30 in-patient beds: two single rooms and four other rooms with capacity for seven patients in each room. These rooms do not have air filters. Infection control methods include surveillance for nosocomial infections and quality control programs targeting hand washing and central line care. All children with febrile neutropenia are admitted and started on broad-spectrum antibiotics. Vancomycin and ceftazidime are used in patients with hypotension or other signs of sepsis. Antifungal coverage is initiated in the case of persistent fever. Overall 80% of blood product requests are met expeditiously; the remaining are met within 24 h.

Abandonment of therapy is a significant treatment challenge in LICs, and efforts to prevent abandonment are an important component of care. In El Salvador, social workers contact families who abandon therapy by telephone. When this is not possible, local health providers visit the patient's home and community to encourage the resumption of treatment. As our study focused on TRM rather than all causes of treatment failure, patients who abandoned treatment were censored in the analysis because they were no longer at risk for TRM. In studies of other clinical outcomes, abandonment is generally treated as an event, with a corresponding decrement in the EFS when a patient abandons treatment.

### Outcome measures

Treatment-related mortality was defined as death unrelated to refractory or progressive disease occurring before first remission was achieved, or any death in complete remission. Death in complete remission was defined as any death occurring 42 days or later after diagnosis in patients who achieved remission. These definitions are consistent with those used by others (Christensen *et al*, 2005; Slats *et al*, 2005) to facilitate future comparisons across studies.

All cases of TRM were also sub-divided by specific cause of death into four categories as follows: infection, bleeding, organ failure, and metabolic (predominantly tumour lysis syndrome). Non-TRM causes of death (i.e., death from progressive disease) also were recorded.

### Potential predictors

Several variables were examined as potential predictors of TRM. These were categorized as biological, socioeconomic, and nutritional. Biological variables included age, sex, initial cell counts, DNA index (ALL), cell lineage (B precursor *vs* T-cell immunophenotype for ALL), risk stratification (higher *vs* standard for ALL), the and French-American-British classification (M5 *vs* others for AML; Riley *et al*, 1999). Socioeconomic variables included monthly income, cost and time to travel to clinic, number of family members (all continuous variables), parental education (secondary level or greater *vs* primary level or less), and the presence of a household telephone. If educational achievement differed among caregivers within a family, the maximal achievement was considered for the analysis. Nutritional variables included body mass index percentile, triceps skin-fold thickness percentile, and mid-upper arm circumference percentile. Body mass index percentile was calculated relative to the 2000 growth charts published by the Centers for Disease Control and Prevention (Ogden *et al*, 2002). Triceps skin-fold thickness provides a measure of fat mass, whereas the mid-upper arm circumference is a measure of lean mass (Sala *et al*, 2004). These two variables were chosen as they have been previously suggested to be ‘gold standard’ measures of nutritional status (Sala *et al*, 2008).

### Statistical methods

The cumulative incidence of TRM was calculated using the Kaplan–Meier method. For this analysis, relapse and abandonment of therapy were censored as competing events with TRM, which was compared between ALL and AML using the log rank test. The proportion of mortality attributable to treatment in general and to specific causes of death was compared between ALL and AML using the *χ*^2^ test or Fisher's exact test as appropriate. Univariate and multi-variable Cox proportional hazards models were used to explore potential predictors of TRM. Variables with a *P*-value <0.1 on univariate analysis were examined in the multiple regression models. Statistical analyses were performed using SAS-PC software (version 8.0; SAS Institute, Cary, NC, USA) or the Statistical Package for Social Sciences for Windows (version 10.1; SPSS, Chicago, IL, USA). Statistical significance was defined as *P*<0.05. The study was approved by the research ethics committees at The Hospital for Sick Children and the Benjamin Bloom National Children's Hospital.

## Results

In the time frame of interest, 547 patients were treated on the protocols, and all were included in the analysis; 469 had ALL and 78 had AML. Demographic data are shown in Table 1. Two- and 5-year EFS rates were 67.7±2.4 and 47.3±3.5% for ALL and 28.3±5.6 and 23.6±5.6% for AML (*P*<0.0001). Two- and 5-year overall survival rates were 79.8±2.1 and 61.1±3.7% for ALL and 34.9±6.3 and 26.8±6.3% for AML (*P*<0.0001). The 2-year cumulative incidence of TRM was 12.5±1.7% for ALL and 35.1±6.4% for AML (*P*<0.0001).

There were 107 deaths in the ALL group and 47 in the AML group (Table 2). The proportion of these deaths attributable to treatment was similar to children with ALL (51.4%) and AML (53.2%, *P*=0.98). Similarly, there was no difference in the proportion attributable to the four most common causes of TRM (infection, bleeding, organ failure, and metabolic) by diagnosis.

**Figure 1:**
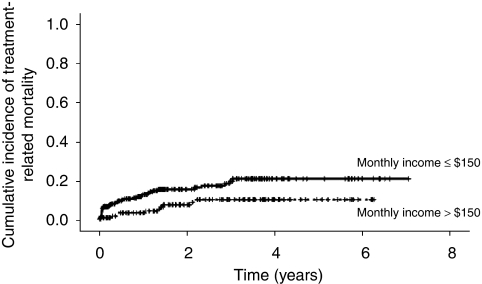
Cumulative incidence of treatment-related mortality in ALL stratified by monthly family income >$150 or ⩽$150.

**Figure 2:**
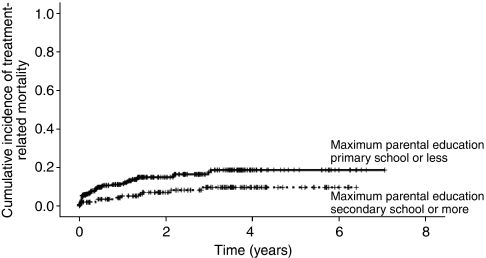
Cumulative incidence of treatment-related mortality in ALL stratified by parental education.

Table 3 shows the univariate analyses examining predictors of TRM. Among patients with ALL, lower monthly income (hazard ratio (HR) per $100=0.81; 95% CI: 0.66, 0.99; *P*=0.04, Figure 1) and lower parental education (HR=0.46, 95% CI: 0.23, 0.89; *P*=0.02, Figure 2) significantly increased the risk of TRM. For children whose maximum parental educational achievement was at least secondary school level, the 2-year cumulative incidence of TRM was 7.1±2.3%. For children whose parental maximal educational achievement was less than the secondary school level (i.e., primary school or illiterate), the 2-year cumulative incidence of TRM was 15.0±2.3% (*P*=0.02). Both higher initial white blood cell count (HR per 10 × 10^9^ cells/l=1.00, 95% CI: 1.00, 1.00; *P*=0.05) and non-B precursor immunophenotype (HR=0.53, 95% CI: 0.26, 1.09; *P*=0.08) were non-significantly associated with increased risk of TRM.

As monthly income and parental education were highly correlated (Spearman's correlation coefficient=0.46, *P*<0.0001), they could not be examined in the same multiple regression model. When each of monthly income and parental education were placed in multivariable models with initial white blood cell count and B-precursor immmunophenotype, both remained independently predictive of TRM (monthly income adjusted HR per $100=0.81, 95% CI: 0.66, 0.99; *P*=0.04; higher parental education adjusted HR=0.47, 95% CI: 0.24, 0.91; *P*=0.03). In both multivariable models, neither initial white blood cell count nor immunophenotype remained independently predictive of TRM.

Among patients with AML, neither biological, socioeconomic, nor nutritional measures were associated with TRM (Table 3).

Table 4 illustrates our estimates of TRM relative to those shown in HICs and other LICs.

## Discussion

Despite significant reductions in treatment intensity, more than 10% of children with ALL and about one-third of children with AML die of TRM in El Salvador, which constitutes about half of the deaths in each disease. Finally, lower socioeconomic status is associated with higher TRM in ALL but not in AML.

Our estimates are consistent with those reported in other LICs (Table 4). However, our report is the first to compare the relative contribution of TRM with treatment failure for children with ALL and AML in an LIC. In HICs, TRM accounts for more treatment failure in children with AML than ALL (Christensen *et al*, 2005; Ravindranath *et al*, 2005; Slats *et al*, 2005; Creutzig *et al*, 2006; Pui and Evans, 2006). Our finding suggests that in LICs, efforts to reduce TRM are as important for children with ALL as for those with AML.

Our study is also unique, as we were able to examine socioeconomic and nutritional variables and their impact on TRM in addition to the traditional biological and clinical factors. Although several studies have examined predictors of TRM in HICs (Wheeler *et al*, 1996; Riley *et al*, 1999; Creutzig *et al*, 2004; Rubnitz *et al*, 2004; Christensen *et al*, 2005; Slats *et al*, 2005), very few have looked at similar risk factors in LICs (Advani *et al*, 1999). Among patients with ALL in HICs, studies have identified gender, age, high white blood cell count, high-risk ALL, T-ALL, and Down's syndrome as TRM risk factors, although these have not been consistent (Wheeler *et al*, 1996; Rubnitz *et al*, 2004; Christensen *et al*, 2005; Slats *et al*, 2005). Conversely, Advani *et al* (1999) found that in the LIC of India, low haemoglobin, lymphadenopathy, and malnutrition were all associated with increased risk of TRM in ALL. They theorised that underlying population biological features as well as more advanced stage at presentation may change the underlying relationships between clinical parameters and TRM.

For children with ALL in El Salvador, in contrast to the above-cited reports in both HICs and LICs, we found that biological factors were not associated with TRM in multiple regression models, and that only poorer socioeconomic status independently predicted TRM. More specifically, only lower household income and lower parental education were associated with higher risk of TRM in ALL. These socioeconomic variables may be proxies for several factors, some of which have been proposed to explain the outcome gap between HICs and LICs (Howard and Wilimas, 2005). These factors include more advanced stage due to delayed presentation and diagnosis, underlying malnutrition, abandonment of treatment (censored in this analysis), delayed recognition of treatment-related complications, or decreased ability to seek prompt medical attention. Initial white blood cell count, measures of malnutrition, and the time and cost of travelling to clinic were not significantly associated with TRM in our study, therefore suggesting that delayed presentation, malnutrition, and distance from the treating facility were not important contributors to TRM in El Salvador. Further research is therefore required to better understand the pathways through which socioeconomic status exerts its negative effect on outcome in ALL.

These findings have implications for future interventions aimed at reducing TRM. Patients of lower socioeconomic status may benefit from more aggressive supportive care in hospital. However, the significant role of socioeconomic status in predicting TRM suggests that further improvements to supportive care may not be as effective in patients with childhood leukaemia as interventions targeting pre-hospital care. Educational efforts aimed at helping parents recognise treatment-related complications may have the greatest impact.

In AML within HICs, female gender, age, high white blood cell count, low performance status at treatment initiation, M5 histology, and the central nervous system status have all been identified as increasing the risk for TRM, although again these findings have varied significantly from study to study (Riley *et al*, 1999; Creutzig *et al*, 2004; Rubnitz *et al*, 2004; Slats *et al*, 2005). A recent study examining children with AML enrolled on a Children's Cancer Group clinical trial found that age >16 years, non-white ethnicity, and underweight status were all significantly associated with infection-related mortality (Sung *et al*, 2007). We did not find that malnutrition influenced TRM in our study. The difference in the effect of malnutrition between the Children's Cancer Group trial and our data may be related to the differences in treatment, underlying differences in study populations, or lack of power in our study.

Strengths of this study include the population-based nature of its data source; there is only one paediatric oncology treatment centre in El Salvador whose patients are all captured by POND. Another is our ability to examine numerous socioeconomic and nutritional variables in addition to clinical and biological factors. Finally, active community follow-up in cases of abandonment of treatment ensures that cases of mortality are not missed or misclassified. Our report is limited by the relatively small number of children with AML available, thus limiting power in this cohort. In addition, the POND database does not capture those patients who died before reaching the central treatment centre.

Treatment-related mortality was higher in AML than in ALL in this LIC; however, the proportion of mortality attributable to treatment was similar. Socioeconomic factors predicted the risk of TRM within ALL. Future work should include efforts to understand the various pathways through which socioeconomic status may affect TRM and to design and evaluate interventions to reduce TRM in ALL. Multi-centre studies across Central America are needed to better study TRM in AML.

## Figures and Tables

**Table 1:** Patient demographic characteristics by diagnosis

**Characteristic**	**ALL**	**AML**
Age in years, median (range)	5.2 (0.0, 15.1)	5.5 (0.2, 15.7)
Male, *N* (%)	263 (56)	40 (51)
Initial WBC (10^9^l^−1^) median (IQR)	11.5 (4.5, 36.3)	15.8 (6.7, 56.6)
Initial haemoglobin (g l^−1^), median (IQR)	69 (51, 90)	77 (50, 97)
		
*Immunophenotype, no (%)*		
B lineage	421 (92)	
T lineage	35 (8)	
		
*FAB classification,* N *(%)*		
M0		7 (9)
M1		18 (24)
M2		26 (34)
M4		8 (11)
M5		6 (8)
M6		2 (3)
M7		7 (9)
		
*Risk group,* N *(%)*		
Higher risk	194 (44)	
Standard risk^a^	250 (56)	
		
*Parental education,* N *(%)*		
Illiterate	17 (4)	3 (4)
Primary	290 (63)	51 (67)
Secondary	107 (23)	18 (24)
Advanced	41 (9)	3 (4)
Unknown	4 (1)	1 (1)
		
Hours to travel to clinic, median (IQR)	2.0 (1.0, 3.0)	2.0 (1.0, 3.0)
Monthly household income (dollars), median (IQR)	150 (100, 250)	150 (57, 228)
Body mass index percentile, median (IQR)	43.2 (11.1, 81.3)	41.2 (10.7, 77.8)
Triceps skin-fold thickness percentile, median (IQR)	17.5 (2.5, 62.5)	37.5 (17.5, 62.5)
Mid-upper arm circumference percentile, median (IQR)	17.5 (2.5, 37.5)	17.5 (2.5, 37.5)

Abbreviations: ALL=acute lymphoblastic leukaemia; AML=acute myeloid leukaemia; FAB=French-American-British; IQR=interquartile range; N, number; WBC, white blood cell count; .

a Standard risk was defined as DNA index ⩾1.16, WBC <50 × 10^9^/l, age >12 months and <10 years, without any of the following: (1) T-cell lineage, (2) CNS leukaemia at diagnosis, (3) testicular leukaemia or (4) bone marrow M3 on day 15 or M2-M3 on day 36.

**Table 2:** Percentage of mortality attributable to treatment and specific causes of death stratified by diagnosis

	**ALL**	**AML**	***P*-value**
Total number of deaths	107	47	
			
*Morality attributable to*			
Treatment, *N* (%)	55 (51.4)	25 (53.2)	0.98
Disease, *N* (%)	52 (48.6)	22 (46.8)	
			
*Specific causes of TRM*			
Infection	38 (70.4)	14 (60.9)	0.4
Bleed	10 (18.5)	7 (30.4)	
Organ failure	3 (5.6)	2 (8.7)	
Metabolic	3 (5.6)	0	

Abbreviations: ALL=acute lymphoblastic leukaemia; AML=acute myeloid leukaemia; TRM=treatment-related mortality.

**Table 3:** Univariate analysis of biological, socioeconomic, and nutritional variables as predictors for TRM stratified by diagnosis

	**ALL**			**AML**		
	**HR**	**(95% CI)**	***P*-value**	**HR**	**(95% CI)**	***P*-value**
*Biological variables*						
Age (years)	0.93	(0.85, 1.02)	0.13	0.99	(0.89, 1.11)	0.90
DNA index	0.57	(0.20, 1.63)	0.29			
B-precursor immunophenotype	0.53	(0.26, 1.09)	0.08			
High-risk group	0.86	(0.50, 1.49)	0.60			
FAB class (M5 *vs* other)				1.95	(0.57, 6.66)	0.29
Initial WBC (per 10 × 10^9^/l)	1.00	(1.00, 1.00)	0.05	1.00	(1.00, 1.00)	0.31
Initial haemoglobin (g l^−1^)	0.98	(0.91, 1.07)	0.70	1.01	(0.87, 1.17)	0.88
						
*Socioeconomic variables*						
Hours to clinic	0.97	(0.84, 1.11)	0.61	1.01	(0.73, 1.39)	0.96
Cost to travel to clinic	1.02	(0.93, 1.11)	0.72	1.08	(0.79, 1.50)	0.62
Income (per $100)	0.81	(0.66, 0.99)	0.04	1.04	(0.89, 1.22)	0.61
Presence of telephone	0.75	(0.41, 1.36)	0.34	1.04	(0.41, 2.68)	0.93
Number of family members	0.94	(0.80, 1.09)	0.39	0.98	(0.81, 1.18)	0.82
Parental education category	0.46	(0.23, 0.89)	0.02	0.94	(0.36, 2.42)	0.89
						
*Nutritional variables*						
Body mass index percentile	1.00	(0.99, 1.02)	0.42	1.00	(0.99, 1.02)	0.87
Triceps skin-fold thickness percentile	1.00	(0.98, 1.02)	0.86	1.02	(0.98, 1.06)	0.29
Mid-upper arm circumference percentile	1.01	(0.99, 1.03)	0.38	1.03	(1.00, 1.06)	0.06

Abbreviations: ALL=acute lymphoblastic leukaemia; AML=acute myeloid leukaemia; BMI=body mass index; CI=confidence interval; HR=hazard ratio; TRM=treatment-related mortality; WBC=white blood cell count.

**Table 4:** Various rates of TRM in ALL and AML in both HICs and LICs

**Reference**	**Protocol**	**HIC *vs* LIC**	**Country**	**TRM (%)**
*ALL*				
Christensen *et al* (2005)	NOPHO ALL92	HIC	Nordic	3.0
Slats *et al* (2005)	DCOG ALL-6, 7 and 8	HIC	The Netherlands	2.1
Rubnitz *et al* (2004)	Total Therapy XI-XIV	HIC	United States	2.9
Li *et al* (2003)	HKALL 93	HIC	Hong Kong	2.8
Wheeler *et al* (1996)	MRCUKALLX	HIC	United Kingdom	5.6
Gupta S *et al* (current)	St. Jude's Modified	LIC	El Salvador	12.5
Howard *et al* (2004)	Total XI, Total XIII	LIC	Brazil	14.9
Metzger *et al* (2003)	St. Jude's Modified	LIC	Honduras	20.8
Laosombat *et al* (2002)	Modified CCSG	LIC	Thailand	11.5
Advani *et al* (1999)	MCP841	LIC	India	16.0
Aziz *et al* (1997)	Modified BFM	LIC	Pakistan	14.3
				
*AML*				
Creutzig *et al* (2006)	AML-BFM 98	HIC	Various	7.2
Ravindranath *et al* (2005)	POG 8821	HIC	Various	14.4
Slats *et al* (2005)	DCOG AML-82, 87 and 92/94	HIC	Netherlands	16.6
Creutzig *et al* (2004)	AML-BFM 93	HIC	Various	9.7
Rubnitz *et al* (2004)	AML-83, 87, 91, 97	HIC	United States	7.6
Riley *et al* (1999)	UKMRC AML10	HIC	Various	13.8
Gupta S *et al* (current)	Modified BFM93	LIC	El Salvador	33.1
Dluzniewska *et al* (2005)	PPLLSG 98	LIC	Poland	18.0

Abbreviations: ALL=acute lymphoblastic leukaemia; AML=acute myeloid leukaemia; HIC=high-income countries; LIC=low-income country; TRM=treatment-related mortality.
